# The Impacts of the COVID-19 Pandemic on Hong Kong Nursing Students’ Mental Health and Quality of Life

**DOI:** 10.3390/ijerph192215117

**Published:** 2022-11-16

**Authors:** Maria Shuk Yu Hung, Winnie Wing Man Ng, Edward Kwok Yiu Choi

**Affiliations:** 1 School of Health Sciences, Caritas Institute of Higher Education, Hong Kong, China; 2 Division of Science, Engineering and Health Studies, College of Professional and Continuing Education, The Hong Kong Polytechnic University, Hong Kong, China; 3 Chinese Language Education and Assessment Centre, Lingnan University, Hong Kong, China

**Keywords:** fear, emotional states, quality of life, nursing students, mental health, COVID-19

## Abstract

Evidence shows that university students, especially healthcare students, experienced considerable health impacts during COVID-19. This study examined Hong Kong general nursing students’ mental health and quality of life during the COVID-19 pandemic. An online questionnaire composed of personal demographics, the Fear of COVID-19 scale (FCV-19S), the Depression Anxiety Stress Scale short version (DASS21), and the World Health Organization Quality of Life-BREF (WHOQOL-BREF) was used for data collection in early 2021. Among 380 respondents, 170 (45%) did not attend clinical practicum during the pandemic. Students who did not participate in clinical training scored lower in FCV-19S but higher in WHOQOL-BREF than those who participated (*p* = 0.001 or *p* < 0.001). FCV-19S and WHOQOL-BREF were negatively correlated (r = −0.623 to −0.446, *p* < 0.001). Slight negative correlations were found between the FCV-19S and DASS-21 scores. Although there were no significant differences in DASS21 (*p* = 0.294–0.931) between these two student groups, there was a considerably high prevalence rate of depression (57.1%), anxiety (47.6%), and stress (39.5%). Hong Kong nursing students, especially those who attended clinical practicum during the pandemic, experienced substantial emotional and quality of life implications. Local universities are recommended to organize appropriate interventions to prepare and support nursing students’ wellbeing and health in coping with future disasters.

## 1. Introduction

The COVID-19 pandemic initiated a public health crisis worldwide and continues to have devastating impacts on humans. Globally, more than 625 million people have been infected, and 6.5 million died, as of October 2022 [1]. Various communities and populations’ mental health and quality of life were also severely affected [2,3,4,5,6,7,8,9,10,11]. The literature has reported that university students, females, and the younger population suffered most severely from mental health impacts during the pandemic [3,12,13]. Other evidence showed that university students had the highest prevalence of mental health problems, followed by healthcare professionals and the general population [3,6,12,14]. Corona is a highly contagious pathogenic viral infection, and the World Health Organization (2021) [15] estimated up to 180,000 death of health and care workers before May 2021. Healthcare professionals who cared for suspected or infected clients were under exceptional stress globally and locally [16,17]. Additionally, future healthcare professionals and novice students studying in healthcare programs at universities or colleges with clinical placements might have experienced adverse physical and psychological impacts on their wellbeing during the COVID-19 pandemic. 

## 2. Background Literature

A meta-analysis reviewed 27 studies assessing the psychological effects of COVID-19 among 90,879 college students in 15 countries, and found that 39.4%, 31.2%, 26.0%, 29.8%, and 50.5% of students had anxiety, depression, stress, post-traumatic stress, and impaired sleep quality, respectively [18]. The study reported the adverse psychological effects on university or college students, and highlighted the necessity of establishing appropriate health interventions or strategies to meet their emotional and psychosocial needs [19,20,21]. Nehir and Gungor Tavsanli (2021) found a significant relationship between the fear of COVID-19 and students’ emotional states. The factors affecting emotional states included information about COVID-19, gender, financial status, social assistance, study programs, etc. [19]. Local studies also reflected that university students’ mental health had been remarkably affected by various factors during the pandemic, e.g., teaching and learning modes and fear of getting infected [22,23,24]. University students’ emotional states were moderately affected, and financial burdens and living alone in hostels positively correlated with the students’ adverse psychological conditions [23]. Inadequate information and facilities support from universities for the students’ learning were also reported [24]. 

University or college students in healthcare programs are susceptible to mental health problems [13]. Globally, medical, nursing, and physiotherapy students made indispensable contributions to caring for patients during the pandemic. However, psychological symptoms or distress, such as post-traumatic stress disorder, anxiety, and depression among healthcare students during the pandemic were not uncommon [18,19,20,25,26]. The long timeframe of academic learning and substantial workload, alongside demanding clinical practicums in hospitals facing life and death issues, including the high contagion risk during the COVID-19 pandemic, further intensified the adverse physical and psychological impacts on the wellbeing of healthcare students [27]. Similar situations were found in China. For instance, a survey investigating 7143 China medical college students’ psychological impacts from the COVID-19 pandemic found that 0.9%, 2.7%, and 21.3% had severe, moderate, and mild anxiety, respectively [28]. Additionally, social support was negatively correlated with the level of anxiety (*p* < 0.001), and closely observing college students’ mental health during epidemics was recommended [28]. Li et al. [29] examined 6348 nursing students. They found that 34.97%, 40.22%, and 14.97% had different anxiety levels, depression, and post-traumatic stress symptoms during the pandemic, and suggested appropriate psychological interventions were needed to relieve their adverse psychological effects [29]. 

Guillasper et al., (2021) examined the COVID-19 impact on the quality of life of 345 nursing students in the Philippines [30]. The results demonstrated that quality of life was significantly related to gender and confirmed COVID-19 cases near their home [30]. There was a negative association of resilience with the impact of COVID-19 on the quality of life of nursing students. Another study investigated the relationship between quality of life, resilience, and associated factors among 152 nursing students in rural Appalachia during the COVID-19 pandemic [31]. About one-fifth (21%) to half (54%) of nursing students were recorded as having a poor quality of life. 

The COVID-19 pandemic has caused a global health crisis and chaos in cities, including Hong Kong. By October 2022, there were more than 1.8 million confirmed cases and 10,000 deaths in Hong Kong [32]. Although Hong Kong nursing professionals have had previous experience responding to emerging infectious diseases [33,34,35,36], nursing students are novices or beginners. They have limited knowledge and clinical experience in responding to public health emergencies, including emerging infectious diseases. For the safety of the nursing students, their clinical practicum was temporarily suspended from January to August 2020, and resumed gradually afterward.

As future healthcare professionals, nursing students have responsibilities not only in caring for sick patients in hospitals but also in promoting the physical and mental wellbeing of citizens in the community. Good physical and psychological wellbeing and quality of life are helpful to their professional fundamental caring attributes. Although many overseas studies have investigated the mental health of university students and nursing students, and there have been local studies of university students, there was no study on nursing students’ emotional states during the COVID-19 pandemic. Regarding the research evidence that the COVID-19 pandemic severely affected students, including nursing students, there was a pressing need to understand the mental health of local nursing students. This descriptive cross-sectional study examined the impacts of COVID-19 on Hong Kong undergraduate nursing students. The research question of this study was: ‘What were the mental health impacts and the quality of life among Hong Kong nursing students during COVID-19?’ The objectives were to assess the nursing students’ fear level, emotional states, and quality of life during the pandemic. To address the research objectives, the following hypotheses were tested: (1) COVID-19 had no effect on Hong Kong nursing students’ mental health, and (2) COVID-19 had no effect on Hong Kong nursing students’ quality of life. The results could have implications for local healthcare professionals, faculty in tertiary institutions, and policymakers in creating appropriate policies, strategies, and interventions for supporting nursing students during this pandemic and during similar public health emergencies in the future. 

## 3. Materials and Methods

### 3.1. Design

A quantitative descriptive cross-sectional survey study was organized from September 2020 to June 2021 to examine the impacts of the COVID-19 pandemic on nursing students’ quality of life and mental wellbeing. 

### 3.2. Subjects and Recruitment

Eligible participants were students over 18 years old who studied undergraduate general nursing programs in Hong Kong during the COVID-19 pandemic. Those students studying mental health nursing programs were excluded as their risk of exposure to COVID-19, and the program curriculum, were different. Mass invitation emails were sent to a large healthcare institution with several general nursing programs with more than 2300 undergraduate nursing students. The study information and electronic consent options, along with the online questionnaire link, were distributed to these students. They were encouraged to refer general nursing students in other institutions with appropriate criteria through their online social network or media to join this study.

A sample size of 371 was required for this study. This estimate was based on the proportion of agreement of the Fear of COVID-19 scale (FCV-19S) items. As the proportion (*p*) was unknown, a conservative estimate was set at *p* = 0.5 to produce the most conservative estimate of the sampling error. With a confidence level of 95% and a margin of error of 0.05, a sample size of no less than 385 was required. To adjust for the finite population size of targeted students of 10,000, a sample size of no less than 371 was required. The sample size calculation formula below was used [37]:

$$n=\frac{Z^{2}P\left(1-P\right)}{d^{2}}$$

where

*n* = sample size;
Z = Z statistic for a level of confidence (95% CI, Z = 1.96);
P = expected proportion of agreement (if 50%, P = 0.5);
*d* = precision (if 5%, *d* = 0.05).

### 3.3. Instrument and Outcome Measures

The self-completed online questionnaire consisted of four parts: the participants’ demographics, the Fear of COVID-19 Scale (FCV-19S) [38], the short version of the Depression Anxiety Stress Scale (DASS-21) [39], and the World Health Organization Quality of Life-BREF (WHOQOL-BREF) [40]. The personal demographics included gender and age group, whether they ever attended clinical practicum, and any clinical practicum experience in the past six months during COVID-19, i.e., from August 2020 to January 2021. After the hospitals’ gradual resumption of nursing students’ clinical practicum in late August 2020, students of different years of nursing programs at various tertiary institutions might have been scheduled for clinical practicum arrangements.

The FCV-19S, a seven-item scale, was developed to evaluate the fear of COVID-19 among the general population by Ahorsu’s team [38]. Using a five-item Likert-type scale, the participants designated their agreement level using “strongly disagree” to “strongly agree” statements. The total sum for these seven items ranged from 7–35. Lower scores indicated less fear of coronavirus disease. The DASS-21, composing three domains (depression, anxiety, and stress), was established to examine individual emotional states [39,41]. There is a total of 21 items using a 4-point Likert-type scale ranging from 0 (did not apply to me at all) to 3 (applied to me very much, or most of the time) [39]. The scores for the three negative emotions were calculated by summing the items. The ‘normal’ scores in depression, anxiety, and stress range from 0–9, 0–8, and 0–14, respectively. Higher scores represented more negative emotional states, e.g., mild, moderate, severe, or extremely severe depression, anxiety, or stress. The WHOQOL-BREF was used to assess the quality of life, embracing the physical health, psychological health, social relationships, and environmental domains. It consists of 26 items using a 5-point Likert-type scale. For each quality of life domain we summed the scores and converted them to the final score. Higher total scores indicated a better quality of life [40]. 

These three scales were simple, reliable, and well-validated for studies examining various health and wellbeing perspectives in different countries and populations, including the Chinese [38,39,40,41,42,43]. Approval to use the WHOQOL-BREF was obtained from the WHO, as required. Twenty undergraduate nursing students were invited to join a pilot study with the online questionnaire in mid-January 2021. After receiving feedback, minor adjustments were made to the wording and layout of the online questionnaire.

### 3.4. Ethical Considerations

Before the study implementation, the School Research Committee of Tung Wah College granted ethical approval (Ref No: NUR/SRC/20200125/027). The research study was carried out following the Declaration of Helsinki guidelines. The study information was provided to potential participants, including the aims, risks, benefits, procedures, and voluntary nature of participation, via the online information sheet. Electronic consent was obtained before participation. Participants had the right to refuse to answer any items or withdraw from the study at any time without negative consequences. All the data were kept confidential, and participants’ anonymity was guaranteed. Only the research team members could access the data. The electronic database file will be deleted from the computer after disseminating the research results or destroyed seven years after data collection. 

### 3.5. Data Collection and Analysis

The students were invited to respond to the online survey, and the data were collected from late January to February 2021. A total of 380 undergraduate nursing students from different tertiary institutions responded to the online survey. After collecting the online questionnaires from participants, descriptive statistics were used to summarize the demographic characteristics, the FCV-19S, WHOQOL-BREF, and DASS-21. Continuous data were expressed as medians (IQRs), as data were not normally distributed. Categorical data were expressed as absolute proportions N (%). For continuous data, the Mann–Whitney U test was used, while categorical data were analyzed by the Pearson Chi-square test or Fisher’s Exact test to determine the statistically significant differences between the participants who attended and did not attend any clinical practicum. Internal consistency of FCV-19S was assessed by McDonald’s omega. Spearman’s rho correlation coefficient was used to investigate the association between two continuous variables. Statistical analysis was performed using IBM SPSS Statistics for Windows, Version 26.0 (IBM Corp., Armonk, NY, USA computer software). A *p*-value of <0.05 was considered statistically significant. 

## 4. Results

### 4.1. Demographic Characteristics

Among the 380 participants, there were more female students (65.5%) than male students (34.5%). The proportion of males in the group “Did not attend any clinical practicum” was 34.4%, significantly lower than in the group “Attended any clinical practicum” (65.6%), *p* = 0.003. Regarding the age groups, the majority (62.6%) were aged between 22 and 25, and 66.8% of the participants in the group “Attended any clinical practicum” were aged 22–25, compared to 33.2% in the group “Did not attend any clinical practicum”, *p* < 0.001. A total of 77.9% of participants were studying a Bachelor Degree in General Nursing, while 2.4% and 19.7% were studying a Master Degree and Higher Diploma in General Nursing, respectively. Please refer to Table 1 for details.

### 4.2. Ratings of FCV-19S, WHOQOL, and DASS-21 between the Groups (Attended and Did Not Attend Any Clinical Practicum)

The McDonald’s omega for the FCV-19S items in Hong Kong subjects was 0.892. The students were asked about their level of agreement with seven statements about COVID-19. All the scores of the seven items of the FCV-19S in the group “Attended any clinical practicum” were higher than the group “Did not attend any clinical practicum”, *p* = 0.001 or *p* < 0.001. The median of the total score of FCV-19S of the group “Attended any clinical practicum” was 25, with an interquartile range of 22.75–28, compared to the median of 21, with an interquartile range of 17–24, for the group “Did not attend any clinical practicum”, *p* < 0.001. The physical health, psychological, and environment scores of the WHOQOL-BREF in the group “Did not attend any clinical practicum” were significantly higher than that in the group “Attended any clinical practicum”, *p* < 0.001. There were no significant differences related to the psychological states of the DASS21 (*p* = 0.294–0.931). Please refer to Table 2 for details.

Remark: The scale for the FCV-19S items was 1—Strongly Disagree, 2—Disagree, 3—Neither Agree nor Disagree, 4—Agree, 5—Strongly Agree. The scale of the DASS-21 was 1—Normal, 2—Mild, 3—Moderate, 4—Severe, 5—Extremely Severe.

### 4.3. Predictors of Total Score of FCV-19S: Regression Analysis

A quantile regression was applied to identify the predictors of the total score of FCV-19S. The potential factors were demographic variables such as gender, age group, type of nursing programme in which students are studying, and whether students attended any clinical practicum. The model showed that younger students and students who attended any clinical practicum were more likely to have higher total scores on the FCV-19S. Please refer to Table 3 for details. 

### 4.4. The Correlations between the FCV-19S, DASS21, and WHOQOL (BREF)

Spearman’s rho correlation coefficient (r) was used to investigate the associations among the FCV-19S (total score), WHOQOL score, and DASS-21 score. The FCV-19S and WHOQOL were found to be significantly negatively correlated, with r = −0.623 to −0.446 and *p* < 0.001. Insignificant negative correlations were found between FCV-19S and DASS-21, with r = −0.062 to −0.016 and *p* > 0.05. Please refer to Table 4 for details.

## 5. Discussion

Hong Kong citizens, including healthcare professionals, have been affected by COVID-19 in physical, psychological, social, environmental, and economic ways [5,10,17,44]. Our study results demonstrated that Hong Kong undergraduate nursing students had high fear levels, negative emotional states, and substantial impacts on quality of life during the COVID-19 pandemic. 

Compared to previous results in the literature, our student participants showed significantly higher levels of depression (57.1%), anxiety (47.6%), and stress (39.5%) symptoms than other local populations [5,44] and college students in Hong Kong, China, or overseas during the pandemic [6,14,18,28]. An online survey of 417 local community women showed that 44.9%, 42.4%, and 32.2% experienced depression, anxiety, and stress, respectively, under the pandemic’s impact [5]. In another study that investigated 500 Hong Kong general public in 2020, 19.8% and 14.0% of the respondents had anxiety and depression, which were lower rates than found in our study results [44]. Li et al. (2021) [6] reviewed and analyzed 27 studies of 706,415 college students’ mental health status, with a prevalence rate of depression and anxiety among them found to be 39% and 36%. Compared to an online survey [22] examining 1648 students at a local university with a similar data collection period (between January to March 2021), our nursing student participants demonstrated higher depression (57.1% vs. 40.0%) and stress (39.5% vs. 22.2%), but similar anxiety (47.6% vs. 50.7%), levels. Our participants’ elevated emotional states, especially among those who had attended clinical practicum, might be related to their nursing professional roles and responsibilities in caring for the sick in hospitals during the COVID-19 pandemic. 

In line with studies conducted in the Philippines [45] and Norway [46], nursing students had high fear levels during the pandemic. Our participants showed high fear levels, with the total median score of the FCV-19S being 23.5, and a more significant score of 25 for those who attended clinical practicum. In a study of 261 Filipino nursing students, the FCV-19S total mean score (for seven items) was 20.34, with the junior nursing students having the highest fear (total mean score = 21.47, SD = 6.49) [45]. In another study of 2605 Norwegian undergraduate nursing students, the FCV-19S item mean score was 2.45 (SD ± 0.8) [46]. Another study evaluated the psychological states of 828 healthcare students in Turkey and found a lower mean FCV19S score of 19.35 (SD ± 5.90) [19]. Our participants’ higher score for the FCV-19S might be due to the perceived risk of the highly contagious Corona, along with local social media news of the upsurge in infected cases and continued overcrowding in hospital admissions [47]. The WHO (2021) [15] estimated that, by May 2021, there would have been up to 180,000 death of health and care workers. In facing such an emerging infectious disease, nursing students’ high fear levels and fear of losing their lives were understandable, as they were beginners in the healthcare profession who did not have experience handling similar situations. The literature showed that the students’ fear of COVID-19 was predominantly related to an insufficient supply of personal protective equipment (PPE), a lack of disaster knowledge and competency in responding to the pandemic [48,49], and the perceived risk of getting infected and transmitting the disease to others or their families [49,50]. 

The present study recognized that students feared getting infected, thinking about Corona, and losing their lives. Indeed, the abrupt increase in face mask and personal protective item usage causing a global shortage in 2020 initiated widespread fears and exaggerated panic worldwide, including in Hong Kong [2,10]. Although this study found insignificant negative correlations between the FCV-19S and DASS-21, the literature indicated that inadequate PPE supply and fear of getting infected significantly correlated with a high anxiety level among nursing students [51]. The students appreciated the opportunity to care for COVID-19 patients in hospitals; however, they were also stressed and afraid [48]. Nursing students thought they were unprepared and incapable of handling complicated clinical situations during the pandemic [52]. However, a sense of commitment and professional obligation supported their participation under the dire threat of the pandemic [53]. Nursing students should be taught how to alleviate their fear and perform self-protection as a priority during clinical placements [52]. De Los Santos et al. (2021) found that the students’ fear of COVID-19 was significantly associated with their readiness and willingness to care for clients who suffered from COVID-19 [45]. Therefore, university teaching faculties and hospital clinical mentors are responsible for teaching nursing students how to protect themselves, ease their fear and anxiety, and respond to challenging clinical situations. 

In addition, this study found a significant difference in fear of COVID-19 and quality of life between those who attended clinical practicum and those who did not. However, there were no significant differences related to emotional states on the DASS21 (*p* = 0.294–0.931) among these two groups. Nursing students might fear contracting the disease and transmitting it to their family, maintain social distance from family members and friends, and worry about the delay in graduation [48,49,54], which may adversely impact their mental health, quality of life, and wellbeing. Furthermore, De Los Santos et al. (2021) [45] found that students’ fear of the COVID-19 pandemic correlated with their intention to quit their nursing studies, which may further aggregate the inadequate professional workforce for the pandemic and future public health emergencies. Indeed, the literature reported that clinical training and duties could initiate different challenges for nursing students and newly graduated nurses on normal days or in chaotic situations induced by disasters [48,49,52,54,55], e.g., the ethical and dignity issues in treating patients, excessive and additional workload, communication, and teamwork in chaotic working conditions.

It is not easy for inexperienced nursing students to cope with such demanding and challenging clinical deployment in times of crisis, as they have little professional experience in responding to emerging infectious diseases and dealing with adverse hospital situations [45,53]. Senior nursing students who joined as healthcare relief volunteers or workers during the pandemic experienced negative emotions due to inadequate professional exposure to highly chaotic situations [53,56]. Griffin and Riley (2022) [57] identified that the major psychological effects on medical and nursing students caused by clinical placement during the pandemic were unfavorable working conditions, adverse effects on participants’ mental health and wellbeing, support from distress, and significant experiences. 

Our study results showed that the participants’ mean scores on the WHOQOL-BREF’s four domains were lower than the WHO reference information (physical = 16.2, psychological = 15.0, social = 14.3, environment = 13.5) [58] and a study of local frontline nurses (physical = 14.75, psychological 14.92, social = 15.21, environmental = 14.48) [17]. This indicated that our participants’ quality of life was affected during the pandemic, including negative emotional states, decreased physical and social activities, and limited and isolated living environments. Hong Kong is a very small urban city which is very densely populated, with more than 7.4 million citizens in living spaces of 16.0 m^2^ per capita [59]. The Government of HKSAR enacted lockdown and social distancing policies in 2020 to reduce the possibility of COVID-19 spread in the community [60]. Most of our participants lived with their parents. With the policy enacted and rising numbers of cases, most healthcare professionals and nursing students preferred to stay in nursing quarters or designated hotel rooms provided/subsidized by their employers after work to minimize the possibility of disease transmission to their families and friends. However, such self-isolation may have further worsened their quality of life. 

In this study, the total score on the FCV-19S and the four different domains of WHOQOL-BREF were found to be significantly negatively correlated (r = −0.623 to −0.446, *p* < 0.001), which indicated the students’ fear of the pandemic adversely affected their quality of life. Results in the current study concurred with previous investigations that nursing students’ fear of COVID-19 affected their quality of life [31,45,61,62], and echoed Ecuadorian research fear of COVID-19 and quality of life among nursing interns, where the respondents had elevated levels of fear which altered their quality of life [61]. Keener and her team (2021) [31] found that nearly half of the nursing student respondents had poor quality of life, and their quality of life was significantly related to their ability and learning. However, Tejoyuwono et al., (2021) [27] reported that with adequate and accurate information about the preventive, control, and treatment measures for COVID-19, the medical students’ emotional states were not impacted, though their quality of life was still affected by the pandemic. 

### 5.1. Implications

Universities and healthcare organizations are urged to provide appropriate strategies and adequate support to ease nursing students’ fear of COVID-19 and future disaster outbreaks, to improve their emotional states and quality of life. Face-to-face or online counseling consultations and mental health service hotlines will be helpful for those who are vulnerable or in need. Pre-clinical workshops could be organized, and particular attention should be given to students before attending clinical practicum in times of an outbreak. Frequent and regular visits by the college academic faculties may promote better understanding of students’ needs and recognition of their challenges in placement venues. Studies highlighted that support from faculties and clinical staff is crucial and could facilitate adaptation and adjustment to clinical processes [50,52]. Organizing mental health workshops, e.g., psychological or mental health first aid training, could help increase nursing students’ mental health self-awareness and ability to offer help to vulnerable peers in need [62,63]. In addition, sufficient resources and support, including staffing, PPEs, and necessary facilities for the admission of infected patients, should be prepared in advance, especially before the situation becomes overwhelming [50]. It should be noted that healthcare professionals and nursing students will be exposed to life-threatening conditions, and their lives and safety should not be overlooked or sacrificed.

The study results highlighted the importance of nursing student training to prepare them to cope with the pandemic and future outbreaks. Practical disaster management training is essential and beneficial to prepare disaster nursing competencies [64]. It could enhance future nurses’ knowledge and skills in coping with public health emergencies and disasters [64,65,66]. Although there is in-service and postgraduate disaster nursing training locally, disaster preparedness training has not been adequately included in the curriculum of undergraduate general nursing training [67]. Evidence shows that simulated disaster education with blended learning activities effectively prepares nursing students’ and improves their confidence in responding to disasters [65,66,68,69]. Therefore, to develop and strengthen future nursing professionals’ disaster competencies, faculties of universities are suggested to integrate more public health emergency and disaster education into the curricula of basic nursing programs. In the long run, promoting healthcare students’ physical and psychological wellbeing will benefit students and will also be beneficial to healthcare professionals, patients, and society.

### 5.2. Limitations

First, the study data were collected through online questionnaires completed by the nursing students, leading to concerns regarding objectivity in self-assessment. Additionally, an online self-reported questionnaire might lead to reporting bias or misunderstanding of the items of the online questionnaire. However, the participants were advised to contact the researchers if concerns arose. The online questionnaire was first disseminated to the nursing students of a local private tertiary institution, and then snowballed to other university nursing students. Although the institution has several nursing programs, the students’ demographics may not be comparable and representative of the student population data in Hong Kong. An appropriate randomized sampling method could be considered in a future study to reduce potential sampling bias.

## 6. Conclusions

The COVID-19 pandemic has enormously impacted various populations, including nursing students, globally. This study revealed that most nursing student participants in Hong Kong were experiencing significant psychological and quality of life implications under COVID-19, especially those who participated in clinical practicum during the pandemic. Local universities and healthcare organizations are encouraged to establish appropriate strategies and activities to sufficiently prepare and support nursing students’ mental health and wellbeing in responding to future public health emergencies and disasters. Comprehensive disaster preparedness and management education are proposed to be included in the curriculum of healthcare-related programs. Future studies aimed at understanding nursing students’ challenges when coping with disasters and evaluating the effectiveness of mental health promotion programs and disaster education are recommended.

## Figures and Tables

**Table 1:** Demographics.

	Did Not Attend Any Clinical Practicum (*n* = 170)	Attended Any Clinical Practicum (*n* = 210)	Overall (N = 380)	
Demographics	*n* (%)	*n* (%)	N	*p*-Value
Gender				* 0.003
Male	45 (34.4%)	86 (65.6%)	131	
Female	125 (50.2%)	124 (49.8%)	249	
Age Group				* <0.001
18–21	83 (77.6%)	24 (22.4%)	107	
22–25	79 (33.2%)	159 (66.8%)	238	
26–29	7 (23.3%)	23 (76.7%)	30	
>30	1 (20.0%)	4 (80.0%)	5	
Which type of nursing programme are you studying?				0.851
Master Degree in General Nursing (Pre-registration)	4 (44.4%)	5 (55.6%)	9	
Bachelor Degree in General Nursing	135 (45.6%)	161 (54.4%)	296	
Higher Diploma in General Nursing	31 (41.3%)	44 (58.7%)	75	

* *p* < 0.05. Categorical variables were analyzed by Pearson Chi-square test or Fisher’s Exact test.

**Table 2:** Ratings of the FCV-19S, WHOQOL, and DASS-21 between the groups (attended and did not attend any clinical practicum).

	Did Not Attend Any Clinical Practicum (*n* = 170)	Attended Any Clinical Practicum (*n* = 210)	Overall (N = 380)	
	Median (IQR)/*n* (%)	Median (IQR)/*n* (%)	Median (IQR)/N (%)	*p*-Value
**Items of FCV-19S**				
F1. I am most afraid of Corona.	4.00 (3.00–4.00)	4.00 (4.00–4.00)	4.00 (4.00–4.00)	* <0.001
F2. It makes me uncomfortable to think about Corona.	4.00 (3.00–4.00)	4.00 (4.00–4.00)	4.00 (3.00–4.00)	* <0.001
F3. My hands become clammy when I think about Corona.	2.00 (2.00–3.00)	3.00 (3.00–4.00)	3.00 (2.00–4.00)	* <0.001
F4. I am afraid of losing my life because of Corona.	3.00 (2.00–4.00)	4.00 (4.00–4.00)	4.00 (3.00–4.00)	* <0.001
F5. When watching news and stories about Corona on social media, I become nervous or anxious.	3.00 (2.00–4.00)	4.00 (4.00–4.00)	4.00 (3.00–4.00)	* <0.001
F6. I cannot sleep because I am worrying about getting Corona.	2.00 (2.00–3.00)	3.00 (2.00–4.00)	3.00 (2.00–4.00)	* <0.001
F7. My heart races or palpitates when I think about getting Corona	2.00 (2.00–3.00)	2.00 (2.00–4.00)	2.00 (2.00–3.00)	* 0.001
Total score of the FCV-19S	21.00 (17.00–24.00)	25.00 (22.75–28.00)	23.50 (19.00–26.00)	* <0.001
**Domains of WHOQOL-BREF**				
WHOQOL-BREF-Physical Health	14.29 (12.00–16.00)	13.14 (11.43–14.29)	13.71 (11.43–15.43)	* <0.001
WHOQOL-BREF-Psychological	12.67 (11.33–14.67)	11.33 (10.67–12.67)	12.00 (10.67–14.00)	* <0.001
WHOQOL-BREF-Social Relationship	14.67 (10.67–16.00)	13.33 (10.67–14.67)	14.00 (10.67–14.67)	0.069
WHOQOL-BREF-Environment	14.00 (11.50–15.00)	12.50 (11.00–13.50)	13.00 (11.00–14.50)	* <0.001
The total score of WHOQOL-BREF	90.00 (75.50–98.00)	79.50 (72.00–87.00)	82.00 (73.00–94.00)	* <0.001
**DASS-21 scales**				
DASS-21—Depression	2.00 (1.00–3.00)	2.00 (1.00–3.00)	2.00 (1.00–3.00)	0.294
Normal	78 (45.9%)	85 (40.5%)	163 (42.9%)	
Mild	24 (14.1%)	38 (18.1%)	62 (16.3%)	
Moderate	49 (28.8%)	52 (24.8%)	101 (26.6%)	
Severe	8 (4.7%)	17 (8.1%)	25 (6.6%)	
Extremely Severe	11 (6.5%)	18 (8.6%)	29 (7.6%)	
DASS-21—Anxiety	1.00 (1.00–3.00)	1.00 (1.00–3.00)	1.00 (1.00–3.00)	0.808
Normal	90 (52.9%)	109 (51.9%)	199 (52.4%)	
Mild	5 (2.9%)	8 (3.8%)	13 (3.4%)	
Moderate	47 (27.6%)	58 (27.6%)	105 (27.6%)	
Severe	15 (8.8%)	11 (5.2%)	26 (6.8%)	
Extremely Severe	13 (7.6%)	24 (11.4%)	37 (9.7%)	
DASS-21—Stress	1.00 (1.00–3.00)	1.00 (1.00–2.00)	1.00 (1.00–2.00)	0.931
Normal	103 (60.6%)	127 (60.5%)	230 (60.5%)	
Mild	22 (12.9%)	34 (16.2%)	56 (14.7%)	
Moderate	32 (18.8%)	22 (10.5%)	54 (14.2%)	
Severe	9 (5.3%)	19 (9.0%)	28 (7.4%)	
Extremely Severe	4 (2.4%)	8 (3.8%)	12 (3.2%)	

* *p* < 0.05. Continuous variables were analyzed by the Mann–Whitney U test.

**Table 3:** Results of quantile regression analysis to predict the total scores of the FCV-19S.

Variables	β	95% CI	*p*-Value
Gender			
Male	0.000	−1.095; 1.095	1.000
Female (reference)	0		
Age Group			
18–21	10.000	5.159; 14.841	* <0.001
22–25	11.000	6.280; 15.720	* <0.001
26–29	11.000	6.031; 15.969	* <0.001
>30 (reference)	0		
Which type of nursing programme are you studying?			
Master’s Degree of General Nursing (Pre-registration)	1.000	−2.692; 4.692	0.595
Bachelor’s Degree of General Nursing	0.000	−1.306; 1.306	1.000
Higher Diploma in General Nursing (reference)	0		
Attended any clinical practicum			
Yes	4.000	2.853; 5.147	* <0.001
No (reference)	0		
Pseudo R2	0.163		

β = unstandardized regression estimates. CI = Confidence Interval. * *p* < 0.001.

**Table 4:** The correlation coefficient (r) among the FCV-19S (total score), WHOQOL, and DASS-21.

	The Total Score of FCV-19S
WHOQOL-BREF-Physical Health	r = −0.557 *p* < 0.001
WHOQOL-BREF-Psychological	r = −0.446 *p* < 0.001
WHOQOL-BREF-Social Relationship	r = −0.473 *p* < 0.001
WHOQOL-BREF-Environment	r = −0.617 *p* < 0.001
WHOQOL-BREF-Total score	r = −0.623 *p* < 0.001
DASS-21-Depression	r = −0.016 *p* = 0.753
DASS-21-Anxiety	r = −0.062 *p* = 0.224
DASS-21-Stress	r = −0.02 *p* = 0.692

The analysis was conducted using Spearman’s rho correlation coefficient.

## Data Availability

The data presented in this study are available on reasonable request from the corresponding author.
